# Sustainable Poly(3-hydroxybutyrate) Bioplastic Production by Extremely Halophilic *Haloarcula* sp. PLQ Isolated from Qatari Extreme Environments

**DOI:** 10.3390/polym18141693

**Published:** 2026-07-09

**Authors:** Manel Ben Abdallah, Imen Saadaoui, Touria Bounnit, Ghamza Al-Ghasal, Mahmoud Thaher, Mohammad A. Al-Ghouti, Nabil Zouari, Helmi Hamdi, Mohamed Chamkha, Sami Sayadi

**Affiliations:** 1 Biotechnology Program, Center for Sustainable Development, College of Arts and Sciences, Qatar University, Doha 2713, Qatar; manelbenabdallah.cbs@gmail.com (M.B.A.); imen.saadaoui@qu.edu.qa (I.S.); touria.bounnit@qu.edu.qa (T.B.); ali88@qu.edu.qa (G.A.-G.); mahmoud.t@qu.edu.qa (M.T.); hhamdi@qu.edu.qa (H.H.); 2 Laboratory of Environmental Bioprocesses, Centre of Biotechnology of Sfax, P.O. Box 1177, Sfax 3018, Tunisia; mohamed.chamkha@cbs.rnrt.tn; 3 Environmental Sciences Program, Department of Biological and Environmental Sciences, College of Arts and Sciences, Qatar University, Doha 2713, Qatar; mohammad.alghouti@qu.edu.qa (M.A.A.-G.); nabil.zouari@qu.edu.qa (N.Z.)

**Keywords:** polyhydroxyalkanoate, poly(3-hydroxybutyrate), biodegradable plastics, Qatari extreme environments, PHB-producing haloarchaea, renewable resources

## Abstract

With the increase in Qatar’s population, the generation of plastic waste has grown, resulting in high levels of environmental pollution. Polyhydroxyalkanoates are sustainable bio-alternatives to petrochemical plastics. Despite their market potential, the industrial implementation of PHAs is still limited. This study aimed to develop sustainable processes for PHA accumulation by screening and isolating novel haloarchaeal strains from Qatari extreme environments with the ability to convert carbon sources to PHAs. In total, 24 positive haloarchaeal members, belonging to *Natrinema*, *Haloarcula*, and *Halostagnicola* genera, were identified for the first time in Qatari ecosystems through 16S rRNA and *phaC*/*phaE* gene sequence analyses. Among them, the promising PHA-producing archaeon *Haloarcula* sp. PLQ exhibited the highest production, reaching a PHB concentration of 496 ± 24 mg L^−1^ and a cell dry weight of 1109.8 ± 58.6 mg L^−1^, corresponding to a maximum yield of 44.69 wt % ± 2.13 under optimal conditions. Polymer characterization confirmed the production of poly(3-hydroxybutyrate). In addition, the thermal properties analyzed by TGA (Tonset = 250 °C; Td = 270 °C) and DSC (Tm = 169 °C) confirmed a PHB-like film with thermal behavior comparable to standard PHB. Therefore, future pilot-scale studies on the pure culture of a promising strain for PHA production from renewable feedstocks under non-sterile, batch, or continuous fermentation will be conducted.

## 1. Introduction

Petroleum-based plastics such as polystyrene (PS), polypropylene (PP), etc., are highly useful materials in our daily lives, and their global production is increasing, currently reaching 413.8 million tons in 2023, of which 58.3% is produced in Asia [1]. Annually, approximately 11.9 million tons of plastic waste is generated in the Gulf co-operation countries (GCC) of which 77% can be incinerated, landfilled, or disposed by open dumping and only 23% is recycled [2]. Significant amounts of plastics are generated in these countries, amounting to 13–14% of total municipal solid waste [3]. With the increase in Qatar’s population, the generation of plastic waste is estimated to amount to 168,000 tons per year, resulting in high environmental impact and serious health risks [4]. In Qatar, littered plastic waste was either allocated in land litter (74%) or in marine litter (26%) [4]. To address this problem, Qatar has adopted the recycling of plastic wastes to enhance waste management. Indeed, only about 40% of the recovered plastic waste is recycled [2]. However, this process alone is not a feasible strategy for waste accumulation, due to the low rates of plastic recycling compared to other materials.

Bioplastics, green alternatives to conventional plastics, have gained increasing attention for use in the pharmaceutical and medical fields as well as in food packaging, due to their properties in terms of biodegradability, biocompatibility, and non-toxicity [5]. Globally, bioplastic production is projected to grow from 2.47 million tons in 2024 to 5.73 million tons in 2029 [6]. Polyhydroxyalkanoates (PHAs) are considered sustainable bio-alternatives to petrochemical plastics including poly(3-hydroxybutyrate) (PHB) and poly(3-hydroxybutyrate-co-hydroxyvalerate) (PHBV). More specifically, PHB is the most extensively studied short-chain-length PHA (scl-PHA) member. This intracellular homopolymer is accumulated by wild-type or genetically recombinant halophiles under unfavorable conditions such as nutrient starvation and carbon excess [7]. PHB materials are stiff, brittle and highly crystalline, exhibiting similar mechanical properties to petroleum-derived plastics [8]. However, industrial PHB production is still limited due to high production costs (around US$4000–15,000 per million tons) and challenges associated with downstream processing for polymer recovery [9]. To overcome these challenges, research efforts are devoted to developing successful processes for cost-effective bioplastic production based on the selection of potent wild-type or recombinant PHB-accumulating strains, efficient carbon substrates, and to optimizing fermentation/recovery processes in order to achieve high yields [10].

Halophilic archaea are promising candidates for high PHA productivity due to several of their unique features: cultivation in high salt concentrations that prevent contamination with limited sterility precautions, the release of intracellular PHAs from the cells with salt-deficient water, and the conversion of inexpensive renewable feedstocks into polyhydroxyalkanoates. Although several haloarchaeal species (around 357, as of December 2023) have been identified [11], only a few haloarchaea have been shown to be excellent biological machines for accumulating PHB. Particularly, members of the genus *Haloarcula* have been investigated for PHB production using renewable resources such as pure sugars [12], biomacromolecules [13], fossil resources [14] and low-cost feedstocks [15]. It was reported that their PHB yields were considerably higher when using pure glucose as the best carbon source, but still low when using low-cost waste substrates in other studies [16]. Therefore, a suitable choice of organic carbon sources and the optimization of operational parameters for fermentation processes are necessary to obtain feasible yields of PHB.

Importantly, certain *Haloarcula* strains possess an enzyme that is responsible for PHA biosynthesis, namely PHA synthase [17,18]. In haloarchaea, PHA synthase is composed of two subunits which form a one-operon *phaEC*. The clustered *phaEC* genes encoding both type III PHA synthase subunits are typical characteristics of PHA-accumulating haloarchaea [19]. Here, this report showed the molecular identification of wild-type PHB-producing haloarchaea targeting 16S rRNA and functional (*phaC*/*phaE*) genes.

To our knowledge, the search for PHB-producing haloarchaea living in extreme environments has been poorly studied. The State of Qatar is considered an arid ecosystem because of its high evaporation rates (>3000 mm/year) and low precipitation rates (<100 mm/year) [20]. Qatari hypersaline sebkhas are also characterized by extreme conditions such as extreme temperature, high salt concentrations and high UV irradiance. However, archaeal members have never been detected in these ecosystems. This is the first comprehensive study in the State of Qatar that is designed to assess the halophilic archaeal diversity with high potential to produce renewable bioplastics in sediments/water samples taken from Ain Al Shamal Al Wardiah, located in Qalaat Al Thaqab. The aim of this study was to search for innovative and eco-friendly processes for bioplastics: polyhydroxyalkanoate (PHA) production, which starts from the screening and isolation of novel potential haloarchaeal strains from harsh Qatari extreme environments as promising converters of carbon sources into PHB. A further aim was to optimize the fermentation conditions/recovery methods and to characterize the polymer extracted from promising strains.

## 2. Materials and Methods

### 2.1. Study Area and Sample Collection

Sampling sites were chosen from two salt lakes (Lake 1 and Lake 2) located in Al Shamal in northern Qatar (Figure S1a,b). Four samples (S1G-24, S2P-24, S3P-24, and S4P-24) were aseptically sampled in the dry season (April 2024), respectively (Figure S1c–f). The S1G-24 and S3P-24 samples, a mixture of salt waters and sediments, were taken in sterile bottles and kept at −80 °C and 4 °C until further processing. The sediments of S4P-24 and the water of S2P-24 have sandy and green colors, respectively, and were also stored at −80 °C and 4 °C. The geographic positions and the physicochemical data of these samples were presented in Table 1. The measurements for pH, electrical conductivity and temperature were made by using a pH/cond 3320 SET 2 (WTW, Germany). The salinity was measured using a seawater Digital Refractometer (BULK REEF SUPPLY).

### 2.2. Isolation of Promising Halophilic PHA Producers

The samples collected from the Qatari lakes were subjected to bioprospecting of archaeal strains with interesting potential to produce PHAs. Hence, aliquots of the samples were inoculated into a PHA-accumulating medium as previously reported [21]. Then, enrichment cultures were incubated at 37 °C, 160 rpm, in an incubator shaker. After 14 days of incubation, a volume of 1 mL was taken from each enrichment culture and serially diluted in 5 mL of liquid medium in each tube followed by spreading 100 µL of each dilution onto the surface of agar plates (2% (*w*/*v*) agar) in duplicate. The solid PHA production medium consisted of the following components (g L^−1^): 250 g NaCl, 10 g MgCl_2_. 6 H_2_O, 15 g MgSO_4_. 7 H_2_O, 4.0 g KCl, 1.0 g CaCl_2_. 2 H_2_O, 0.5 g NaHCO_3_, 1 g yeast extract, 10 g starch, and 2% (*w*/*v*) agar at pH 7. The pH of the medium was adjusted using 1 M HCl or 1 M NaOH. After 10 days of incubation at 37 °C, different colonies were selected and restreaked for several rounds on fresh agar plates and microscopically (ZEISS Primostar 3, Germany) checked for purity (shape/size). The cell motility and morphological characterization were conducted for all the isolates. Their pure cultures were added to a 30% (*v*/*v*) of sterile glycerol as a cryoprotectant for long-term storage in a −80 °C freezer.

### 2.3. Identification of Haloarchaeal Strains

Genomic DNA was extracted from all isolates using a PureLink^TM^ Microbiome DNA Purification Kit (Invitrogen, USA), following the manufacturer’s instructions. Archaeal 16S rRNA genes were amplified using forward 21F (5′-TTCCGGTTGATCCYGCCGGA-3′) and reverse primer 1492R (5′-GGTTACCTTGTTACGACTT-3′), in the same manner as previously described [22]. PCR was performed using an Applied Biosystems^TM^ Veriti^TM^ 96-Well Thermal Cycler (Thermo Fisher Scientific, USA) in a 25 µL reaction volume containing 50 ng of target DNA, 1× PCR Buffer, 2.5 mM MgCl_2_, 0.2 µM of each primer, a 200 µM each DNTP, and 1.25 U of GoTaq^®^ Flexi DNA polymerase (Promega). The following program consisted of one cycle of initial denaturation at 95 °C for 5 min, followed by 30 cycles of 94 °C for 1 min, 55 °C for 1 min, and 72 °C for 2 min, ending with 10 min at 72 °C. The restriction digestion of PCR products using *Hae*III, *Hind*III, *Hinf*I (Life technologies) was carried out as previously reported [22]. 16S rRNA fragments derived from positive producing strains were purified using a GeneJET^TM^ Gel Extraction Kit (Thermo Scientific, Vilnius, Lithuania) and then submitted for sanger sequencing at Weill Cornell Medicine-Qatar. The phylogenetic tree was constructed with MEGA as previously reported [23].

### 2.4. Screening of Potential Halophilic PHA Producers

As previously reported, all isolates were screened for PHA accumulation using specific Nile Red dye (Sigma-Aldrich) [24] and Sudan Black B alcoholic solution (Sigma-Aldrich) [25]. In our previous study, *Natrinema altunense* strain CEJGTEA101 (KY129977) was described as a PHB producer and was used as a positive control. The negative control was represented by the reference bacterial strain *Escherichia coli* DH5α [21].

### 2.5. Screening of Class III PHA Synthase Genes by Degenerate PCR

Genomic DNA of thirty-nine extremely haloarchaeal strains were screened for the detection of class III PHA synthase in their genome using CODEHOP (Consensus-Degenerate Hybrid Oligonucleotide Primer) PCR amplification. As previously reported, the neighboring genes *phaE* and *phaC*, located in *phaEC* operon, were amplified using two pairs of CODEHOPs, codehopEF (forward, 5′-CGACCGAGTTCCGCGAYATHTGGYT-3′)/codehopER (reverse, 5′-GCGTGCTGGCGGCKYTCNAVYTC-3′), and codehopCF (forward, 5′-ACCGACGTCGTCTACAAGGARAAYAARYT-3′)/codehopCR (reverse, 5′-GGTCGCGGACGACGTCNACRCARTT-3′), respectively [21]. PCR was performed using an Applied Biosystems^TM^ Veriti^TM^ 96-Well Thermal Cycler (Thermo Fisher Scientific, USA) in a 25 µL reaction volume containing 50 ng of target DNA, 1× PCR Buffer, 2.5 mM MgCl_2_, 0.2 µM of each primer, a 200 µM each DNTP, and 1.25 U of GoTaq^®^ Flexi DNA polymerase (Promega). The following program for both units *PhaE* and *PhaC* consisted of one cycle of initial denaturation at 94 °C for 5 min, followed by 30 cycles of 94 °C for 30 s, 55 °C for 45 s, and 72 °C for 45 s, ending with 10 min at 72 °C. PCR products purified using a GeneJET^TM^ Gel Extraction Kit (Thermo Scientific, Vilnius, Lithuania) were then sent for sanger sequencing at Weill Cornell Medicine-Qatar. The translation of nucleotide sequences of *phaC* and *phaE* genes into their corresponding amino acid sequences was evaluated in the same manner as previously mentioned [13]. The construction of phylogenetic trees based on amino acid sequences was done as previously indicated [13].

### 2.6. Measurement of Cell Growth and Cell Dry Weight (CDW)

To follow the growth of the best selected PHB halophilic archaeon producer in the PHA-accumulating medium, the absorbance at 600 nm was monitored using a visible spectrophotometer (JENWAY 7310, UK) at regular intervals of 24 h. (2%, (*v*/*v*)) of 96 h old culture was used as inoculum.

To determine the biomass (cell dry weight), 50 mL of late-logarithmic culture was sampled in duplicate and harvested by centrifugation using Megafuge ST plus series centrifuge (Thermo Scientific, USA) at 4752× *g* for 30 min. After washing twice with sterile distilled water, the cell pellets were re-centrifuged, frozen and then lyophilized overnight to reach a constant mass. The freeze-dried cells were used for intracellular PHA recovery and quantification.

### 2.7. Impact of Growth Parameters on PHB Production

In order to optimize PHB accumulation by the selected PHB-producing strain, temperature (30, 37, 40, and 45 °C), salt concentration (100–300 g L^−1^), pH values (6.5, 7, 8, and 9), and incubation times (24–144 h) were monitored. The optimized experiments were tested in duplicate in 50 mL of PHA-accumulating medium amended with starch (10 g L^−1^). The dry biomass and the PHB concentration were measured.

### 2.8. Impact of Carbon Sources on PHB Production

Three pure carbohydrates: starch, glucose, and glycerol, were chosen depending on their availability and cost-effectiveness. D-Glucose, pure glycerol, and soluble starch were purchased from Sigma-Aldrich (Germany). The impact of these carbon sources on PHB production by the selected PHB-producing strain was tested by separately incorporating 1% (*w*/*v*) of each one in PHA-accumulating medium under optimized growth conditions, followed by measuring the dry biomass and the mass of extracted polymer. Glucose, glycerol, and soluble starch were added at 10 g L^−1^, corresponding to a theoretical carbon equivalent of 4, 3.91, and 4.44 g C L^−1^, respectively.

### 2.9. Impact of Nitrogen Sources on PHB Production

Separately, the selected PHB-producing strain was cultivated in PHA-accumulating medium amended with peptone (Suvchem), yeast extract (Gibco), NH_4_Cl (Sigma-Aldrich) or urea (Sigma-Aldrich) at 0.1% (*w*/*v*) under optimized growth conditions. The dry biomass and the mass of extracted polymer were measured.

### 2.10. Polymer Extraction and Quantification

Freeze-dried cells were dissolved in a volume of sodium hypochlorite solution (6%, *v*/*v*)) (LOBA Chemie) equal to the original volume of medium at 37 °C for 1 h to ensure cell lysis. After centrifugation, a white-colored pellet was obtained and consecutively washed with distilled water, acetone (Qualikems, India), and ethanol (Fisher Chemical, UK). The whitish purified pellet was dissolved in chloroform (purity ≥ 99.8%, Sigma-Aldrich, USA). The polymer-containing chloroform phase was poured into a glass beaker and placed in a gas extraction hood until evaporation, resulting in the formation of transparent PHB films [26]. The PHB content (wt %) was determined as the ratio of mass of PHB extracted to mass of dry biomass, multiplied by 100.

### 2.11. Polymer Characterization

#### 2.11.1. Gas Chromatography (GC)

About 4 mg of lyophilized cells were mixed with 1 mL of chloroform, 0.85 mL of methanol, and 0.15 mL of sulfuric acid. After methanolysis (100 °C, 140 min), the PHA content in the freeze-dried cells and its composition were analyzed using a GC-FID (Shimadzu 2010 plus, Japan), equipped with 100 m column, 0.2 um film thickness and 0.25 mm inner diameter (SP2560). The injector temperature was maintained at 240 °C and the detector at 250 °C. Total helium flow was set at 28.9 mL min^−1^. The program was used as follows: 100 °C for 5 min; temperature ramp of 4 °C per min; 240 °C for 10 min, according to the modified method of Brandl et al. [27]. Samples were analyzed in duplicate. Commercially available poly[(R)-3-hydroxybutyric acid] (PHB) (Sigma-Aldrich, USA) was used as a pure standard for calibration. The equation of the calibration curve was as follows:

Y = 2.710287 × 10^−6^ X + 0.354342.

#### 2.11.2. Fourier Transform Infrared Spectroscopy (FTIR)

Spectra were obtained using spectrum 400 FTIR (PerkinElmer, Canada) with an universal attenuated total reflectance accessory in the range of 400 to 4000 cm^−1^ at a spectral resolution of 4 cm^−1^ for eight scans per spectrum [28]. Spectra were examined to deduce the functional groups of the extracted polymer, in comparison with standard PHB.

### 2.12. Thermal Properties of PHB Film

#### Thermogravimetric Analysis (TGA) and Differential Scanning Calorimetry (DSC)

Simultaneous determination of weight loss (TGA) and heat flow (DSC) on the same polymer sample (7.5 mg) was carried out using TA Instruments SDT-Q600 simultaneous TGA/DSC (Artisan Technology Group, IL, USA). The biopolymer sample was exposed to high temperature treatment from 50 to 650 °C under nitrogen gas at a heating rate of 10 °C min^−1^. The thermograms of the extracted polymer have been compared with standard PHB.

## 3. Results and Discussion

### 3.1. Phenotypic and Phylogenetic Characterization of Potential Halophilic PHA Producers

Recent studies have focused on the remarkable potential of haloarchaea in the sustainable production of bioplastics [29]. Therefore, we screened potential halophilic PHA-producing archaea from Qatari extreme environments that have never been investigated and valorized before. These microenvironments are characterized by high variability in salinity and temperature conditions. In addition, the present study revealed physicochemical heterogeneity among sample types, resulting in the growth and survival of halophilic archaeal communities. A total of 39 halophilic PHA-producing archaea were isolated from Qatar’s extreme environments. The collected archaeal strains are used to build the first biobank of archaeal strains in Qatar and the region, which can be exploited for several other biotechnological applications. Colonies in plates after 10 days were round, red, pink, cream, circular, and entire with a diameter between 0.2 and 0.5 mm. Morphological characterization was conducted for all the isolates, revealing the dominance of pleomorphic and cocci forms. The cells are non-motile (Figure S2). Here, 24 PHA-accumulating haloarchaeal isolates were found to be positive, either fluorescent using Nile red dye or black using lipophilic Sudan Black B stain. This useful screening method was widely used for the qualitative observation of PHA producers [30]. Different haloarchaeal strains like *Haloarcula tradensis*, *Natrinema pallidum* [22], and *Natrinema altunense* [21] were rapidly screened by incorporating staining dyes into colonies on agar. Besides colony staining methods, fluorescence microscopy was also used to visualize PHA inclusions in *Haloarcula hispanica* A85, *Natrinema altunense* A112 [31], *Haloarcula* sp. TG1 [18], and *Haloarcula marismortui* [32] cells. The results obtained from positive strains isolated from Lake 1 and Lake 2 are shown in Figure S3 and Figure S4, and Table S1, respectively.

**Figure 1:**
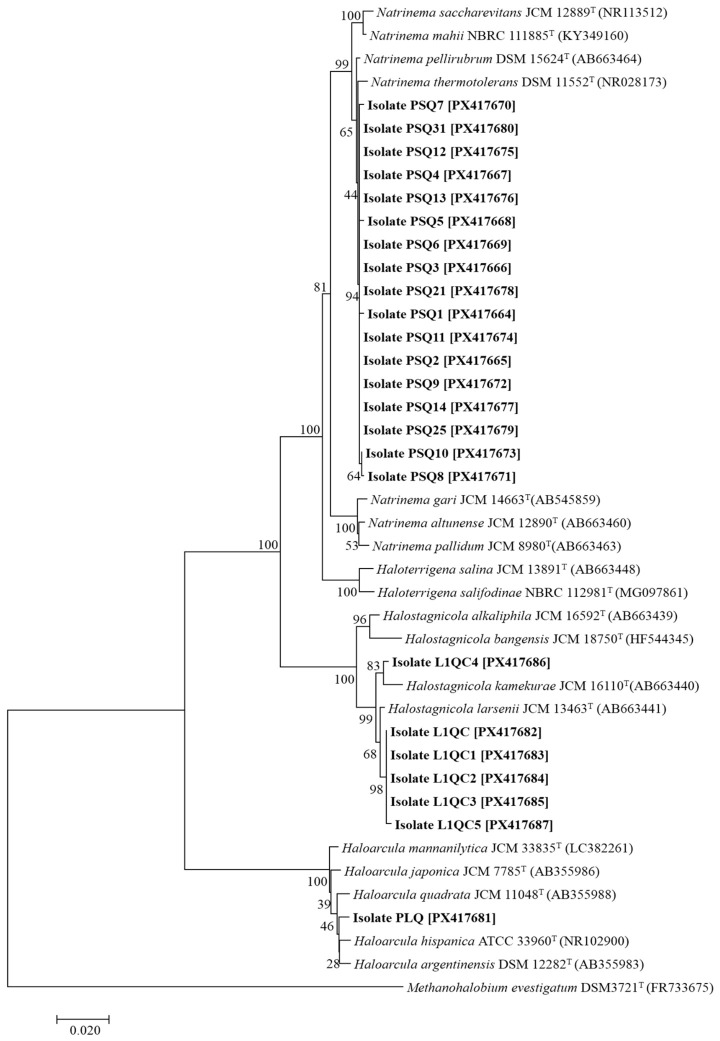
Phylogenetic tree of the PHA-producing haloarchaeal isolates from Ain Al Shamal Al Wardiah targeting 16S rRNA genes. The construction is based on the similarities of our sequences and its relatives using the neighbor-joining method and the Jukes–Cantor model with bootstrapping for 1000 replicates. Scale bar, 0.02 substitutions per site.

The comparison of the ARDRA patterns obtained after digestion of the amplified 16S rRNA gene with the restriction enzymes *Hae*III, *Hinf*I, and *Hind*III revealed different profiles. *Hae*III digestion classified positive producing strains into four restriction patterns, and *Hinf*I into three patterns (Figure S5). However, no restriction fragments were observed on the profiles of *Hind*III digestion. The amplified ribosomal DNA restriction analysis (ARDRA) was used to discriminate between the strains. According to 16S rRNA gene analysis, 24 isolates were found to be positive PHA producers using phenotypic and genotypic tests and were affiliated with *Natrinema*, *Haloarcula*, and *Halostagnicola* genera within the order *Halobacteriales*, class *Halobacteria* and phylum *Methanobacteriota*. The isolate PLQ was related to *Haloarcula quadrata* (99.11% of similarity), *Har*. *japonica* (99.26% of similarity), *Har*. *mannanilytica* (99.2% of similarity), *Har*. *hispanica* (99.2% of similarity), and *Har*. *argentinensis* (99.2%). Seventeen isolates showed close relatedness to species *Natrinema thermotolerans* and *Natrinema pellirubrum*. Finally, the strains L1QC, L1QC1, L1QC2, L1QC3, L1QC4, and L1QC5 were affiliated with the species *Halostagnicola larsenii* (Figure 1).

### 3.2. Molecular Characterization of phaC and phaE Genes in PHA-Producing Cells

**Figure 2:**
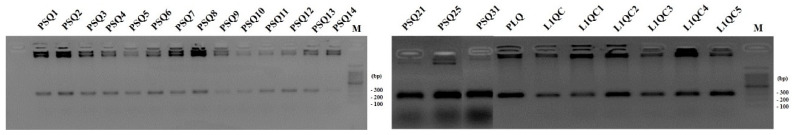
Detection of PHA-producing isolates by PCR amplification of the PHA synthase gene (*PhaC* with approximately 280 bp in size). Lanes M, 100-bp DNA ladder.

**Figure 3:**
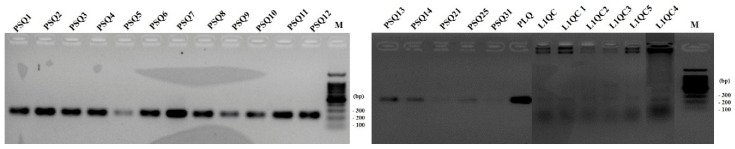
Detection of PHA-producing isolates by PCR amplification of the PHA synthase gene (*PhaE* with approximately 230 bp in size). Lanes M, 100-bp DNA ladder.

Primary screening of PHA producers offers no specificity and can generate false positives. For this reason, molecular methods (including polymerase chain reaction) could be precise and efficient to target the genes encoding for class III PHA synthase. Recently, screening studies involving PCR amplification of the *phaE*/*phaC* genes indicated the detection of PHA synthase in members of the genera *Natrinema* and *Haloarcula* [21,22]. In the present study, genomic DNA of 39 isolates were screened for the detection of the PHA synthase gene in their genome using CODEHOP PCR amplification for PHA synthase genes (type III). The same PHA-producing haloarchaeal isolates, which are described above as positive with phenotypic methods, presented bands of 280 bp (*phaC*) and 230 bp (*phaE*) (Figure 2 and Figure 3). In haloarchaea, PHA synthases are consisted of the structural subunit *phaE* and the catalytic subunit *phaC* [33]. However, few studies based on the molecular identification and genetic characterization of haloarchaeal PHA synthases have been reported [17,18,33,34].

**Figure 4:**
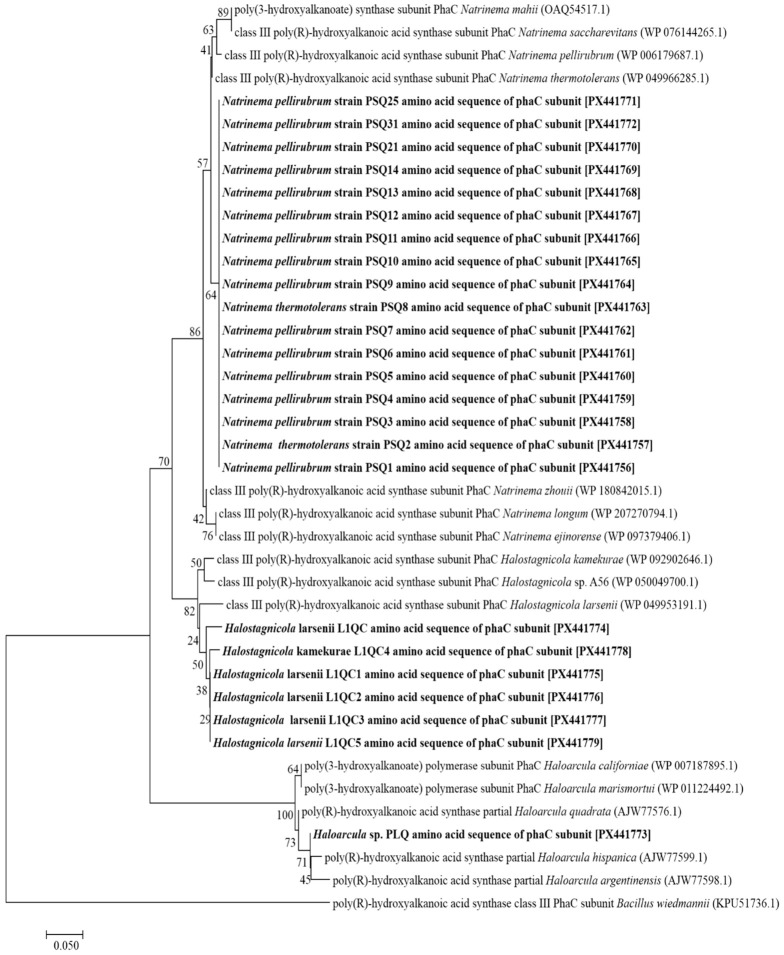
Phylogenetic analysis of amino acid sequences of *phaC* subunit from haloarchaeal genera *Halostagnicola*, *Natrinema*, and *Haloarcula*. The tree was constructed using the neighbor-joining method and the Poisson model with bootstrap analysis on 1000 replicates. Scale bar, 0.05 substitutions per site.

**Figure 5:**
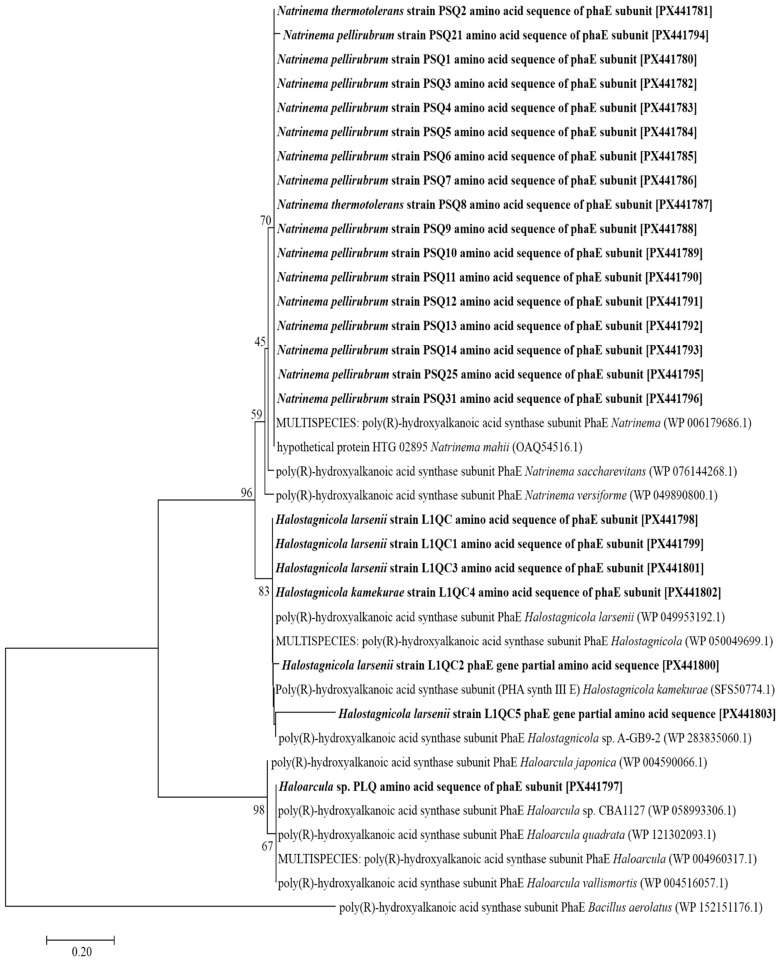
Phylogenetic analysis of amino acid sequences of *phaE* subunits from haloarchaeal genera *Halostagnicola*, *Natrinema*, and *Haloarcula*. The tree was constructed using the neighbor-joining method and the Poisson model with bootstrap analysis on 1000 replicates. Scale bar, 0.2 substitutions per site.

Regarding the corresponding amino acid sequences, the phylogenetic analyses demonstrated that *phaC* from 24 positive PHA-producing isolates showed high levels of identity (99%) with its counterparts from *Haloarcula quadrata*, 97–98.88% with *Natrinema thermotolerans*, and 93.75–95.51% with *Halostagnicola larsenii* (Figure 4). The identities of *PhaE* from positive isolates to those from *Halostanigola larsenii*, *Haloarcula quadrata* and *Natrinema* sp. were 100% identical amino acids (Figure 5). Our results were in agreement with previous findings describing the remarkable potential of PHB-producing haloarchaea to convert carbon sources to PHB, such as *Halobiforma*, *Haloarcula*, *Haloferax*, *Natrinema*, *Halorubrum*, *Natrialba*, *Natronococcus*, *Natronobacterium*, and *Natronomonas* [13,18,33,35]. Interestingly, it was shown that the *phaEC* gene cluster encoding PHA synthase (type III) was identified in *Haloarcula marismortui* [17] and *Haloferax mediterranei* [34]. Han et al. [17] reported the requirement of *phaEC* genes for PHB production and their deletion resulted in a loss of activity of PHA synthase in *Haloarcula* species. In this current study, the detection of *phaE* and *phaC* amino acid sequences as PHB accumulators in *Halostagnicola* sp. was found for the first time.

### 3.3. Optimization of the Fermentation Process Towards Maximum PHB Production in Cells of Haloarcula sp. PLQ

**Figure 6:**
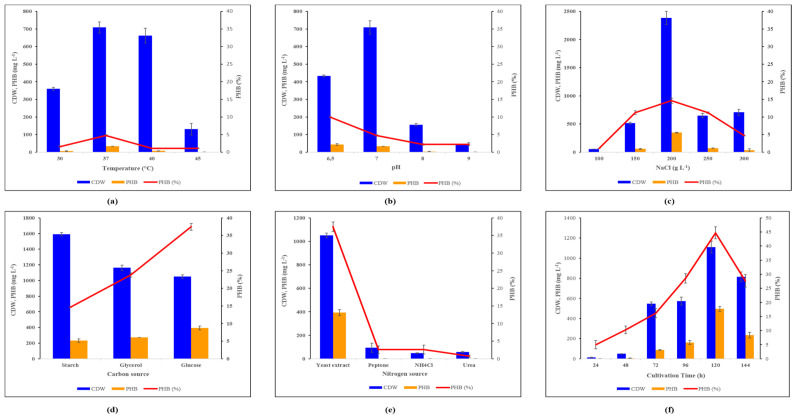
Optimization of PHB production in the cells of *Haloarcula* sp. PLQ using a number of factors: Temperature (**a**), pH ranges (**b**), NaCl concentrations (**c**), Carbon sources (**d**), Nitrogen sources (**e**), and Cultivation times (**f**). The data are represented as the mean of two replicates ± standard error. PHB contents in dried cells were obtained using the extraction method (biomass digestion with sodium hypochlorite), as described above.

To the best of our knowledge, several halophilic archaea, especially *Haloferax mediterranei*, were able to accumulate PHBV from various pure carbohydrates including starch, glucose, and glycerol [36]. Despite their increasing number, there are limited reports on halophilic archaea producing homopolymer PHB in their cells from structurally unrelated carbon sources or carbon-rich wastes [15]. In the current study, promising PHB-producing haloarchaea were isolated from Qatari extreme environments. These isolates were affiliated with *Haloarcula*, *Natrienema* and *Halostagnicola* genera. Despite the wide distribution of members of *Natrinema* in hypersaline environments, these potential PHB producers are firstly described in Qatari lakes. Additionally, members of *Haloarcula*, which have been recovered from different hypersaline environments, have previously been reported to be good producers of PHB as well as hydrocarbon degraders [16,37]. On the other hand, this report indicated that the *Halostagnicola* isolates were considered putative PHA producers harbouring *phaC*/*phaE* genes. Among them, 24 positive PHA producers were subjected to quantitative primary screening through gas chromatography. The quantitative analysis showed that the strain PLQ exhibited the highest cell dry weight (1525 ± 5 mg L^−1^) and PHA production (205.8 ± 36 mg L^−1^), yielding a PHA recovery rate of 13.5 ± 2.31%. Therefore, optimization of cultivation conditions for maximum PHB production should be considered. Based on the unique ARDRA pattern, fluorescence intensity and GC findings, the extremely halophilic archaeon *Haloarcula* sp. PLQ was found to be the best positive strain and was selected for further studies. Here, the optimization experiments were done in duplicate to evaluate the impact of temperature (30–45 °C), pH (6.5–9), sodium chloride concentration (100–300 g L^−1^), incubation period (24–144 h), carbon/nitrogen type on cell dry weight (CDW) estimation and PHA production by the strain PLQ (Figure 6). These cultivation parameters were also optimized in other studies to achieve a higher PHA concentration [18,38]. In parallel, the curve profiles of the promising strain PLQ at different fermentation parameters were also investigated in PHA-accumulating medium as described in the Section 2 (Figure S6). This strain grew at a temperature range of 30–45 °C with an optimum temperature of 37 °C (Figure S6a). Figure 6a illustrated that the strain PLQ reached 708.3 ± 31.7 mg L^−1^ of maximum CDW, containing 33.6 ± 0.4 mg L^−1^ of maximum PHB at 37 °C. At extreme temperatures, there was a decrease in the growth of *Haloarcula* sp PLQ; however, no PHAs were recovered. This strain was then cultivated in a pH range between 6.5 and 9 at 37 °C. The highest cell-dried biomass and PHB concentration were recorded at 37 °C and pH 7.0, with a low recovery yield of 4.75 ± 0.2% (*w*/*w*) (Figure 6b). It was reported that members of the genus *Haloarcula* are aerobic, extremely neutrophilic haloarchaea with an optimum between 6.5 and 7.5 [39]. As shown in Figure 6c, CDW, PHB concentration and its yield increased significantly at a sodium chloride concentration of 200 g L^−1^, 37 °C, pH 7.0, reaching 2383.2 ± 116.8 mg L^−1^, 348 ± 17 mg L^−1^ and 14.6 ± 0.72% (*w*/*w*), respectively. Below 10% NaCl, there was no biomass or detectable PHB production due to cell lysis at low salt concentration. Additionally, the effect of varying carbon sources on growth and PHB production of the selected strain was evaluated. The replacement of starch with the same concentration (10 g L^−1^) of pure sugars such as glycerol or glucose in PHA-accumulating medium increased the PHB content to 23.8 ± 0.6% or 37.47 ± 1%, respectively (Figure 6d). When using starchy substrate as the sole carbon source, the lyophilized biomass rate was higher (an average of 1590.5 ± 21.7 mg L^−1^); however, the PHB production was lower compared to other substrates (an average of 14.6 ± 0.05% of CDW). This might be explained by slow consumption of starch. These findings were similar to those reported by Kurt-Kızıldoğan et al. [18]. I PHB contents in the dried cells of *Haloarcula* strains were considered significantly higher than those in CDW of *Haloarcula* sp. PLQ in the case of using soluble starch as the sole carbon sources [12,13,18]. Applying pure glycerol, the strain PLQ attained CDW of 1000 ± 34.2 mg L^−1^ and a PHB production of 272 ± 0.1 mg L^−1^ after 120 h, yielding a PHB content of 23.8 ± 0.6% of its CDW. Hermann-Krauss et al. [40] reported that *Haloferax mediterranei* produced 16.2 g L^−1^ of P-3(HB-co-10%-HV) using crude glycerol, slightly higher than the value obtained from pure glycerol (13.4 g L^−1^). This is one of the few studies focused on PHB production using crude/pure glycerol by employing haloarchaeal species. When grown on glucose and yeast extract, PHB production increased with fermentation duration (range from 67 ± 7 to 393 ± 25 mg L^−1^), reaching a maximum recovery yield of 37.47 ± 1% (*w*/*w*) at 120 h in PHA-producing medium supplemented with 10 g L^−1^ of glucose as easily assimilated substrate and 1 g L^−1^ of yeast extract as the best nitrogen source (Figure S7d). When compared to other studies, the PHB productions by *Haloarcula japonica*, *Haloarcula hispanica*, and *Haloarcula marismortui* using glucose as the sole carbon source were 0.5%, 2.4% and 21%, much lower amounts than those obtained in this present study [17,41,42]. Under optimal cultivation conditions, the promising strain PLQ was cultivated for different periods in a PHA-accumulating medium containing 10 g L^−1^ glucose plus 1 g L^−1^ yeast extract at 37 °C; PH 7.0; and 20% NaCl (Figure 6f). After a lag phase of 2 days, the culture of *Haloarcula* sp. PLQ attained an exponential growth phase of 3 days and reached the stationary phase after 120 h of incubation (Figure S6e). Interestingly, an increase in DCW and PHB generation until 120 h then a sharp decline during the stationary phase were observed (Figure 6f). It seems that the maximum cell dry weight (1109.8 ± 58.6 mg L^−1^) and maximum PHB concentration (496 ± 24 mg L^−1^), corresponding to a maximum yield of 44.69 ± 2.13% of its CDW, were recorded at the early stationary phase after 120 h of incubation. The volumetric productivity under the optimized condition was approximately 4.13 mg L^−1^ h^−1^. The results of the parameters used to analyse the intracellular PHB production in *Haloarcula* cells under optimized conditions are shown in Table S2.

### 3.4. Characterization of PHB Film

**Figure 7:**
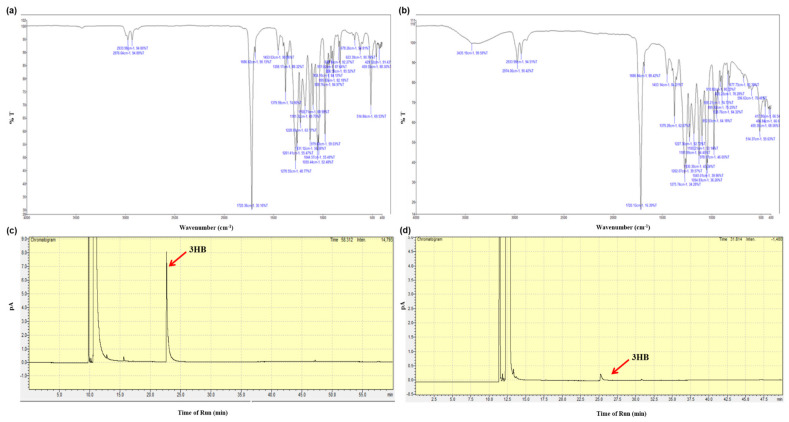
FTIR spectra of (**a**) standard PHB (Sigma-Aldrich), (**b**) PHB produced by *Haloarcula* sp. PLQ grown in PHA-accumulating medium supplemented with 10 g L^−1^ glucose and 1 g L^−1^ yeast extract; Chromatograms of (**c**) standard PHB (Sigma-Aldrich), (**d**) PHB produced by *Haloarcula* sp. PLQ grown in PHA-accumulating medium supplemented with 10 g L^−1^ glucose and 1 g L^−1^ yeast extract.

The chemical structure of the extracted polymer from *Haloarcula* sp. PLQ was analyzed by means of FTIR spectroscopy. The spectra of the extracted polymer were compared with those of the commercial PHB, showing the bonding interactions present in the sample film (Figure 7a,b). The spectra of biodegradable film and standard PHB showed a characteristic peak at 1720 cm^−1^, corresponding to carbonyl stretching vibration (C=O), as previously reported [43]. Prominent bands at 1055, 1130, and 1276 cm^−1^ related to carboxyl group (C-O) were also observed [44]. It was shown that the transmittance region from 2800–3100 cm^−1^ represents C-H stretching [45]. Here, weak peaks at 2934 and 2974 cm^−1^ were assigned to the C-H stretching bond, indicating the presence of methylene (-CH_2_-) and methyl (-CH_3_) groups, respectively. In addition, the peak at 1454 cm^−1^ is attributed to asymmetric bending of –CH_3_ [18]. The weak peak at 3435 cm^−1^ corresponded to the free O-H group [46]. The FTIR spectra of purified film recovered from *Haloarcula* sp. PLQ grown on glucose as the best carbon source was very similar to what was reported [13] and matched well with those of the standard PHB. All the major peaks at 2974, 2934, 1276, 1130, 1055, 979, and 515 were found to be present for short-chain-length PHA (scl-PHA), corresponding to poly(3-hydroxybutyrate) [47].

The composition of isolated polymer was also analyzed by means of gas chromatography. The spectrum of standard PHB exhibited an intense peak at a retention time of 24 min (Figure 7c). Similarly, the chromatogram of the 3-hydroxybutyrate methylester obtained from the extracted polymer revealed a weak peak with retention time of 25 min using the 100 m SP-2560 capillary column (Figure 7d). This retention time is longer than those reported in previous studies employing shorter columns, where the resulting 3HB-methylester (C-4) appeared at around 4–5 min [27]. The variation of retention times in gas chromatography can be explained by several factors including the type of GC column used, the temperature program, the carrier gas flow rate, etc. On the other hand, no peaks corresponding to 3-hydroxyvalerate (3HV) methylester were detected using GC-FID and FTIR analyses, suggesting that PHB was the predominant polymer produced. However, this does not exclude the possibility of 3HV being present at levels below the detection limit of the analytical method or the inability of the strains to synthesize PHBV under the tested conditions.

**Figure 8:**
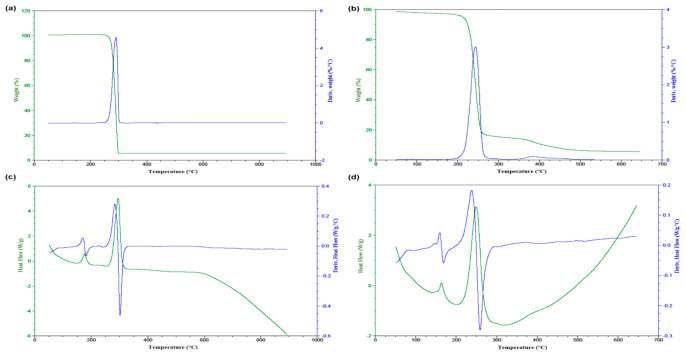
TGA thermograms of (**a**) standard PHB (Sigma-Aldrich), (**b**) PHB produced by *Haloarcula* sp. PLQ grown in PHA-accumulating medium supplemented with 10 g L^−1^ glucose and 1 g L^−1^ yeast extract; DSC thermograms of (**c**) standard PHB (Sigma-Aldrich), (**d**) PHB produced by *Haloarcula* sp. PLQ grown in PHA-accumulating medium supplemented with 10 g L^−1^ glucose and 1 g L^−1^ yeast extract.

The thermal properties of the extracted polymer were studied by means of TGA and DSC analyses. There are very few reports on TGA and DSC characterization of the investigated polymers produced by halophilic archaea. In this current study, the thermal stability was analyzed by TGA. The thermograms derived from standard PHB showed a sharp decrease in the curve, indicating a rapid single-stage decomposition. The onset temperature of the standard PHB was 250 °C and the maximum rate degradation temperature (T_max_) ranged from 290 to 310 °C (Figure 8a). With respect to temperature, the derivative weight curve showed a prominent peak at 290 and 310 °C, which corresponds to the maximum rate decomposition temperature of standard PHB and represents a one-step thermal degradation process. No secondary peaks were observed, thus confirming the high purity of PHB. In this study, the thermograms derived from the extracted polymer from *Haloarcula* sp. PLQ showing the mass curves and their derivatives as functions of temperature are reported in Figure 8b. The weight of the sample film remained stable until about 240–250 °C (T_onset_). The TG curve profile of the sample film showed that it is a thermally stable in a temperature ranging from 50 °C to 250 °C. At approximately 250 °C (T_onset_), an initial decomposition of the extracted polymer occurred. The major step of its weight loss fell from 100% to 20% in the range 250–300 °C with a maximum decomposition temperature (T_d_) of 270 °C. Similarly, Soni et al. [48] reported that the thermal degradation of PHB produced by *Haloarcula* sp. AB19 isolated from salt pans ranged from 260 to 340 °C. Here, the TG curve of our sample continued with weight losses, until reaching a T_d_ 10% of 500 °C. Beyond 500 °C, a weight loss of 90% is observed, showing that the polymer sample has largely decomposed. Recently, Kurt-Kızıldoğan et al. [18] reported a maximum thermal decomposition of PHB produced by *Haloarcula* sp. TG1 at 498.4 °C. Here, the derivative curves indicated a main peak at 250 °C and smaller fluctuations, which correspond to some residual inorganic content, similar to other reports [49]. Compared to standard pure PHB, the TG curve profile matched well with the commercial PHB and revealed an excellent thermal stability of the extracted PHB from *Haloarcula* sp. PLQ.

The DSC analysis was performed to assess the thermal transitions of the extracted polymer when the sample was heated. The DSC graphs of the polymer sample produced by extremely archaeon *Haloarcula* sp. PLQ utilizing glucose and the standard PHB are shown in Figure 8c,d. Here, it was found that the first peak, corresponding to the melting temperature of the polymer sample, was about 169 °C (T_m_) as mentioned in the profile of standard PHB. A second endothermic peak in the heat flow curve occurred at 270 °C. These results may be compared with those of Hassan et al. [50], who showed a first peak at T_m_ 176 °C and a second peak at T_m_ 299 °C for PHB produced by the Azhar strain of *Bacillus subtilis* using glucose as a carbon source. Furthermore, DSC thermograms of extracted PHB from the strain *Haloarcula* sp. AB19 revealed that the melting temperature (T_m_) varied between 170 and 174 °C [48]. On the other hand, a higher T_m_ value of PHB extracted from *Haloarcula* sp. TG1 was observed [18]. It is evident that the structural and thermo-mechanical characterization of the PHB materials depend on the choice of species, carbon sources, growth conditions, and extraction methods [5]. The TGA, DSC, FTIR, and GC findings confirmed that the biopolymer synthesized by extremely haloarchaeon *Haloarcula* sp. PLQ was identified to be poly(3-hydroxybutyrate).

### 3.5. Morphological Characterization of PHB Film

**Figure 9:**
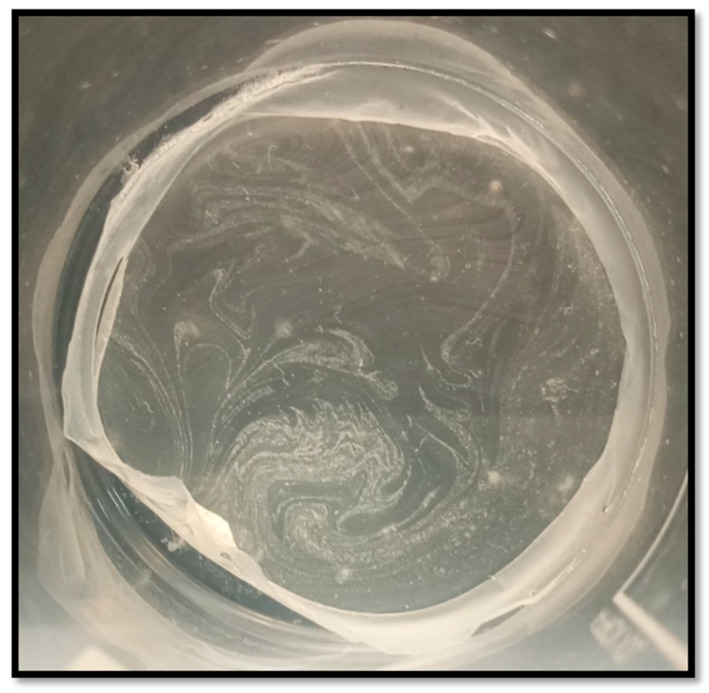
PHB film obtained by *Haloarcula* sp. PLQ under optimal cultivation conditions using the solvent evaporation method.

The polymer produced by extremely halophilic archaeon *Haloarcula* sp. PLQ under optimal cultivation conditions was used to prepare film by means of the solvent evaporation method. Following chloroform evaporation, the film obtained was transparent, colorless, and rigid (Figure 9).

## 4. Conclusions and Perspectives

On the basis of the data obtained, a wide diversity of PHA-accumulating archaea were described in Qatari extreme environments, never having been investigated before. What is notable is that *phaC*/*phaE* genes encoding PHA synthase (type III) are widespread in our haloarchaeal positive PHA producers. Among them, extremely halophilic archaeon *Haloarcula* sp. PLQ exhibited promising results in terms of dried cells (1109.8 ± 58.6 mg L^−1^), and intracellular PHA accumulation (44.69 ± 2.13%). This value represents the intracellular PHA content rather than the substrate-to-product conversion yield. This value is considerably higher than that reported in other lab-scale experiments. The polymer extracted was found to be poly(3-hydroxybutyrate). Therefore, it would be interesting to conduct studies on PHB production in pilot-scale by means of the pure culture of our promising strain *Haloarcula* sp. PLQ using renewable feedstocks and to analyze its whole-genome sequencing. Further investigation of substrate utilization kinetics, residual substrate concentration, and carbon conversion efficiency will be necessary to strengthen industrial process evaluation. Interestingly, this report indicated that the *Halostagnicola* isolates were considered putative PHA producers harboring *phaC*/*phaE* genes. Taking this into account, it is important to further investigate quantitative PHB production in *Halostagnicola* cells, optimize the fermentation process, and characterize the purified polymer in future studies.

## Figures and Tables

**Table 1:** Environmental parameters of samples and distribution of halophilic isolates from Al Shamal Lakes.

Sample Type	Sample	Site	Lake	Latitude	Longitude	T (°C)	pH	CE (mS/cm)	Salinity (g L^−1^)	Total Number of Isolates
Water and sediments	S1G-24	Site 1	Lake 1	26°03′68.8″ N	51°11′0.67″ E	23.6	8.15	9.15	6.93	6
Water	S2P-24	Site 2	Lake 2	26°02′25.5″ N	51°06′48.8″ E	23.8	8.195	17.54	15	1
Water and sediments	S3P-24	Site 3	Lake 2	26°02′25.5″ N	51°06′48.8″ E	23.9	8.179	19.1	16.23	11
Sediments	S4P-24	Site 4	Lake 2	26°02′25.5″ N	51°06′48.8″ E	24.8	7.98	74	62.9	21

## Data Availability

The archaeal 16S rRNA sequences were submitted to the GenBank database under accession numbers PX417664 to PX417687. The nucleotide sequences of *phaC* and *phaE* genes received the accession numbers PX441756 to PX441779 and PX441780 to PX441803, respectively.

## Supplementary Materials

The following supporting information can be downloaded at: https://www.mdpi.com/article/10.3390/polym18141693/s1. Figure S1: Panoramic views of investigated samples collected from Al Shamal Lakes. (a) Lake 1, (b) Lake 2, (c) Sample S1G-24 (Lake 1), (d) Sample S2P-24 (Lake 2), (e) Sample S3P-24 (Lake 2), (f) Sample S4P-24 (Lake 2); Figure S2: Phase contrast microscopy images of PHA-producing cells; bar, 10 µm; Figure S3: Cells accumulating PHA staining with Nile Red (a) and Sudan Black B (b) on agar plates for the strains isolated from the sample S1G-24 (Lake 1). Control +: The PHB-producer *Natrinema altunense* strain CEJGTEA101. Control −: The reference bacterial strain *Escherichia coli* DH5α; Figure S4: Cells accumulating PHA staining with Nile Red (a,c,e) and Sudan Black B (b,d,f) on agar plates for the strains isolated from the sample S4P-24 (Lake 2) except the strain PLQ (sample S2P-24). Control +: The PHB-producer *Natrinema altunense* strain CEJGTEA101. Control −: The reference bacterial strain *Escherichia coli* DH5α; Figure S5: ARDRA profiles obtained by digestion of amplified 16S rRNA of PHA-producing isolates with restriction enzymes *Hae*III (A) and *Hinf*I (B). Lanes M, 1 Kb DNA marker; Figure S6: Time course of growth of the isolate *Haloarcula* sp. PLQ at different temperatures (a), pH ranges (b), NaCl concentrations (c), carbon sources (d), and nitrogen sources (e); Figure S7: Effect of optimized fermentation parameters on dry cell weight and PHB production of *Haloarcula* sp. PLQ. The data are represented as the mean of two replicates ± standard error. Table S1: Screening of PHA-producing archaeal strains using phenotypic and genotypic methods; Table S2: Results of the parameters used to analyze the intracellular PHB production in *Haloarcula* cells under optimized conditions.
