# Supplementary material for: Sustainable Poly(3-hydroxybutyrate) Bioplastic Production by Extremely Halophilic Haloarcula sp. PLQ Isolated from Qatari Extreme Environments
Source: Polymers (Basel). 2026 Jul 9;18(14):1693. doi: 10.3390/polym18141693 (PMC13431313; doi:10.3390/polym18141693)
Supplement: Supplementary file 1 [file polymers-18-01693-s001.zip › polymers-4315475-supplementary.pdf]

Article

Sustainable Poly(3-hydroxybutyrate) Bioplastic Production by Extremely Halophilic *Haloarcula* sp. PLQ isolated from Qatari Extreme Environments

Manel Ben Abdallah [1,2,*], Imen Saadaoui [1,*], Touria Bounnit [1], Ghamza Al-Ghasal [1], Mahmoud Thaher [1], Mohammad A. Al-Ghouti [3], Nabil Zouari [3], Helmi Hamdi [1], Mohamed Chamkha [2] and Sami Sayadi [1,*]

[1] Biotechnology Program, Center for Sustainable Development, College of Arts and Sciences, Qatar University, Doha 2713, Qatar; manelbenabdallah.cbs@gmail.com (M.B.A.); imen.saadaoui@qu.edu.qa (I.S.); touria.bounnit@qu.edu.qa (T.B.); ali88@qu.edu.qa (G.-G.); mahmoud.t@qu.edu.qa (M.T.); hhamdi@qu.edu.qa (H.H.); ssayadi@qu.edu.qa (S.S.)
[2] Laboratory of Environmental Bioprocesses, Centre of Biotechnology of Sfax, BP 1177, 3018 Sfax, Tunisia; mohamed.chamkha@cbs.rnrt.tn (M.C.)
[3] Environmental Sciences Program, Department of Biological and Environmental Sciences, College of Arts and Sciences, Qatar University, Doha P.O. Box 2713, Qatar; mohammad.alghouti@qu.edu.qa (M.A.-G.); nabil.zouari@qu.edu.qa (N.Z.)
* Correspondence: ssayadi@qu.edu.qa (S.S.); manelbenabdallah.cbs@gmail.com (M.B.A.); imen.saadaoui@qu.edu.qa (I.S.); Tel.: +974 3157 6288 (S.S.)

Captions

Figure S1. Panoramic views of investigated samples collected from Al Shamal Lakes. (**a)** Lake 1, (**b**) Lake 2, (**c**) Sample S1G-24 (Lake 1), (**d**) Sample S2P-24 (Lake 2), (**e**) Sample S3P-24 (Lake 2), (**f**) Sample S4P-24 (Lake 2).

Figure S2. Phase contrast microscopy images of PHA-producing cells; bar, 10 µm.

Figure S3. Cells accumulating PHA staining with Nile Red (**a**) and Sudan Black B (**b**) on agar plates for the strains isolated from the sample S1G-24 (Lake 1). Control +: The PHB-producer *Natrinema altunense* strain CEJGTEA101. Control -: The reference bacterial strain *Escherichia coli* DH5α.

Figure S4. Cells accumulating PHA staining with Nile Red (**a**, **c**, **e**) and Sudan Black B (**b**, **d**, **f**) on agar plates for the strains isolated from the sample S4P-24 (Lake 2) except the strain PLQ (sample S2P-24). Control +: The PHB-producer *Natrinema altunense* strain CEJGTEA101. Control -: The reference bacterial strain *Escherichia coli* DH5α.

Figure S5. ARDRA profiles obtained by digestion of amplified 16S rRNA of PHA-producing isolates with restriction enzymes *Hae*III (A) and *Hinf*I (B). Lanes M, 1 Kb DNA marker.

Figure S6. Time course of growth of the isolate *Haloarcula* sp. PLQ at different temperatures (**a**), pH ranges (**b**), NaCl concentrations (**c**), carbon sources (**d**), and nitrogen sources (**e**).

Fig. S7 Effect of optimized fermentation parameters on dry cell weight and PHB production of *Haloarcula* sp. PLQ. The data are represented as mean of two replicates ± standard error.

Supplemental Figures

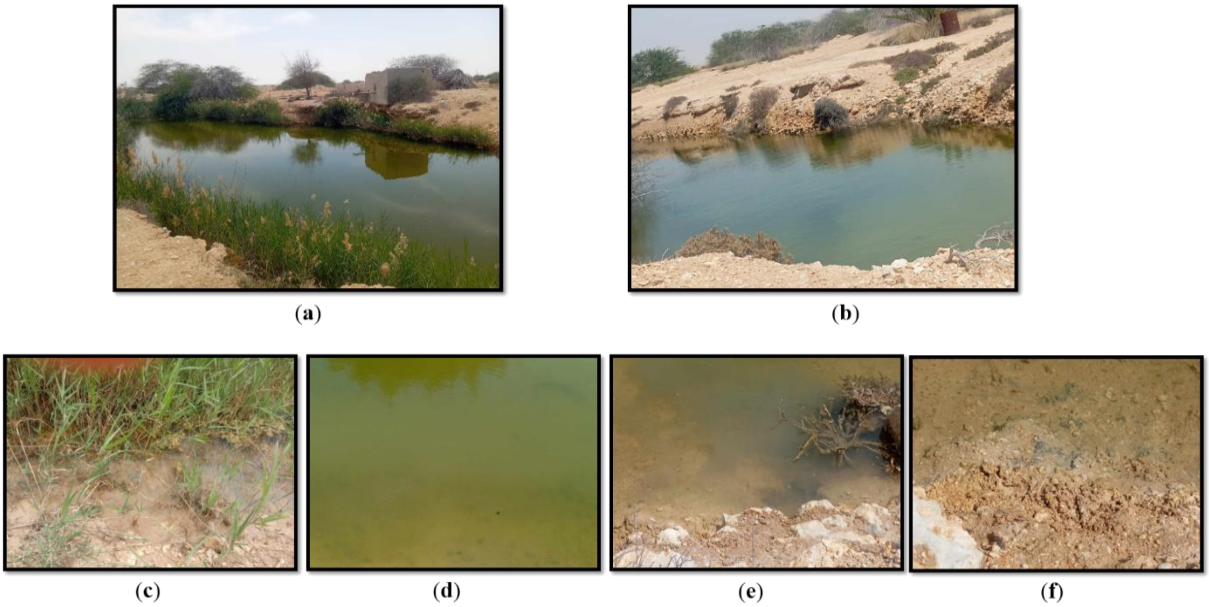

Figure S1. Panoramic views of investigated samples collected from Al Shamal Lakes. (**a)** Lake 1, (**b**) Lake 2, (**c**) Sample S1G-24 (Lake 1), (**d**) Sample S2P-24 (Lake 2), (**e**) Sample S3P-24 (Lake 2), (**f**) Sample S4P-24 (Lake 2).

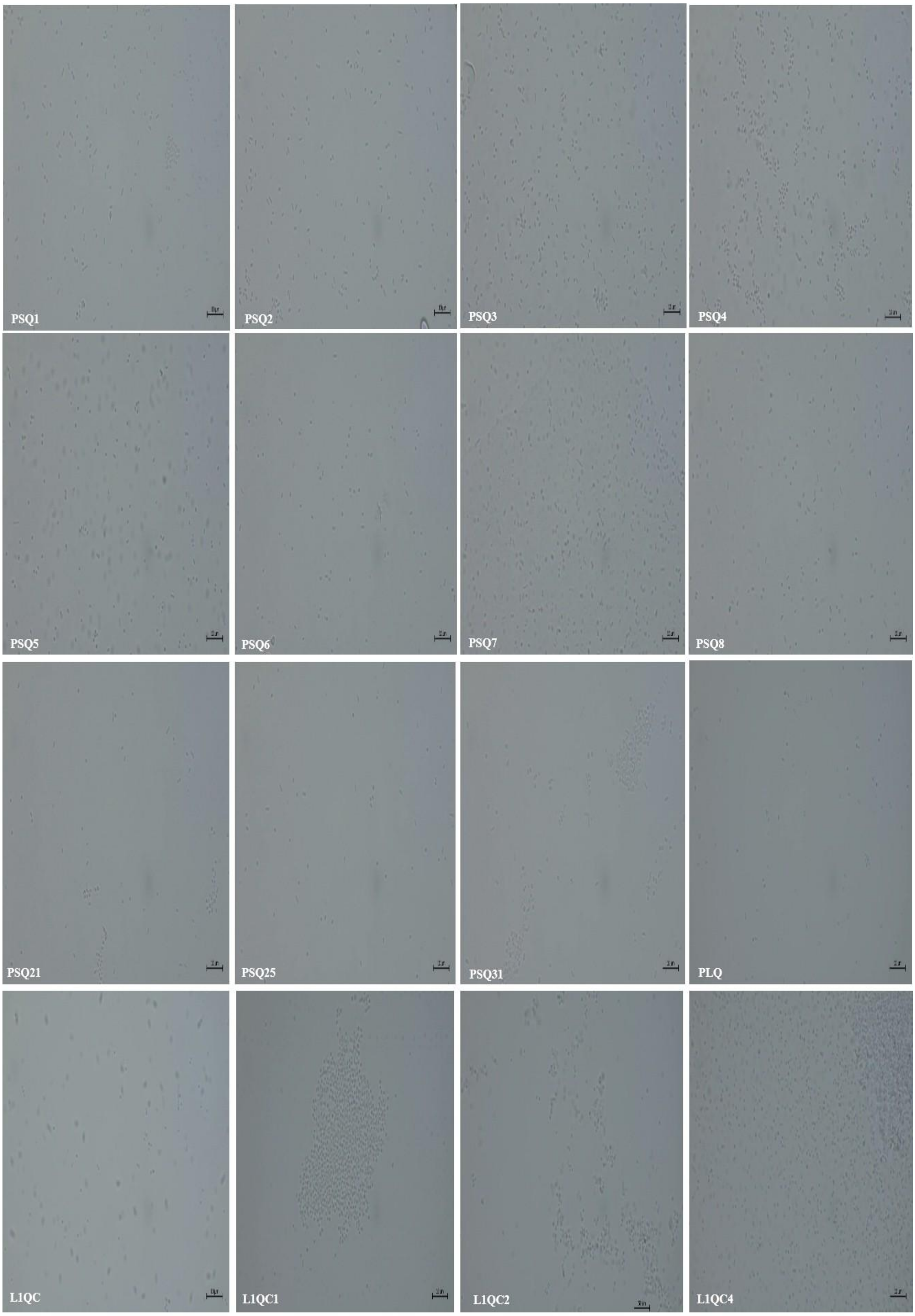

Figure S2. Phase contrast microscopy images of PHA-producing cells; bar, 10 µm.

a

b

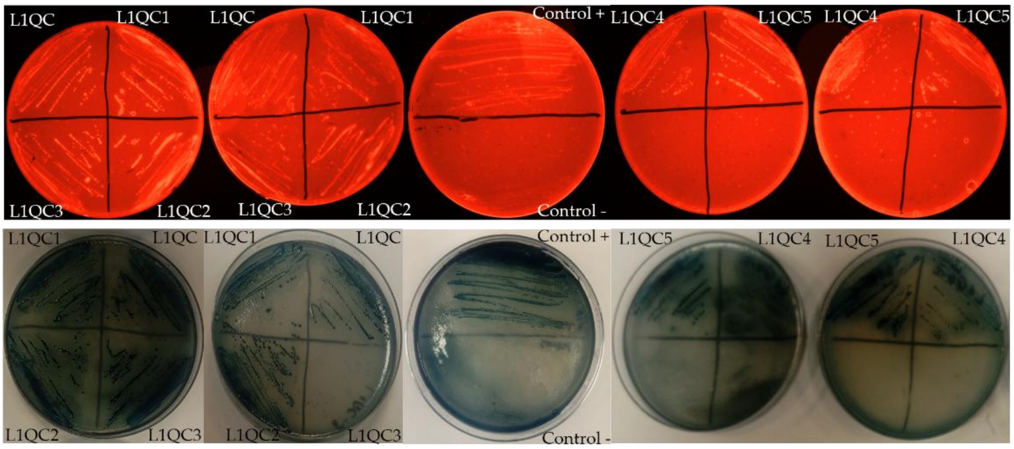

Figure S3. Cells accumulating PHA staining with Nile Red (**a**) and Sudan Black B (**b**) on agar plates for the strains isolated from the sample S1G-24 (Lake 1). Control +: The PHB-producer *Natrinema altunense* strain CEJGTEA101. Control -: The reference bacterial strain *Escherichia coli* DH5α.

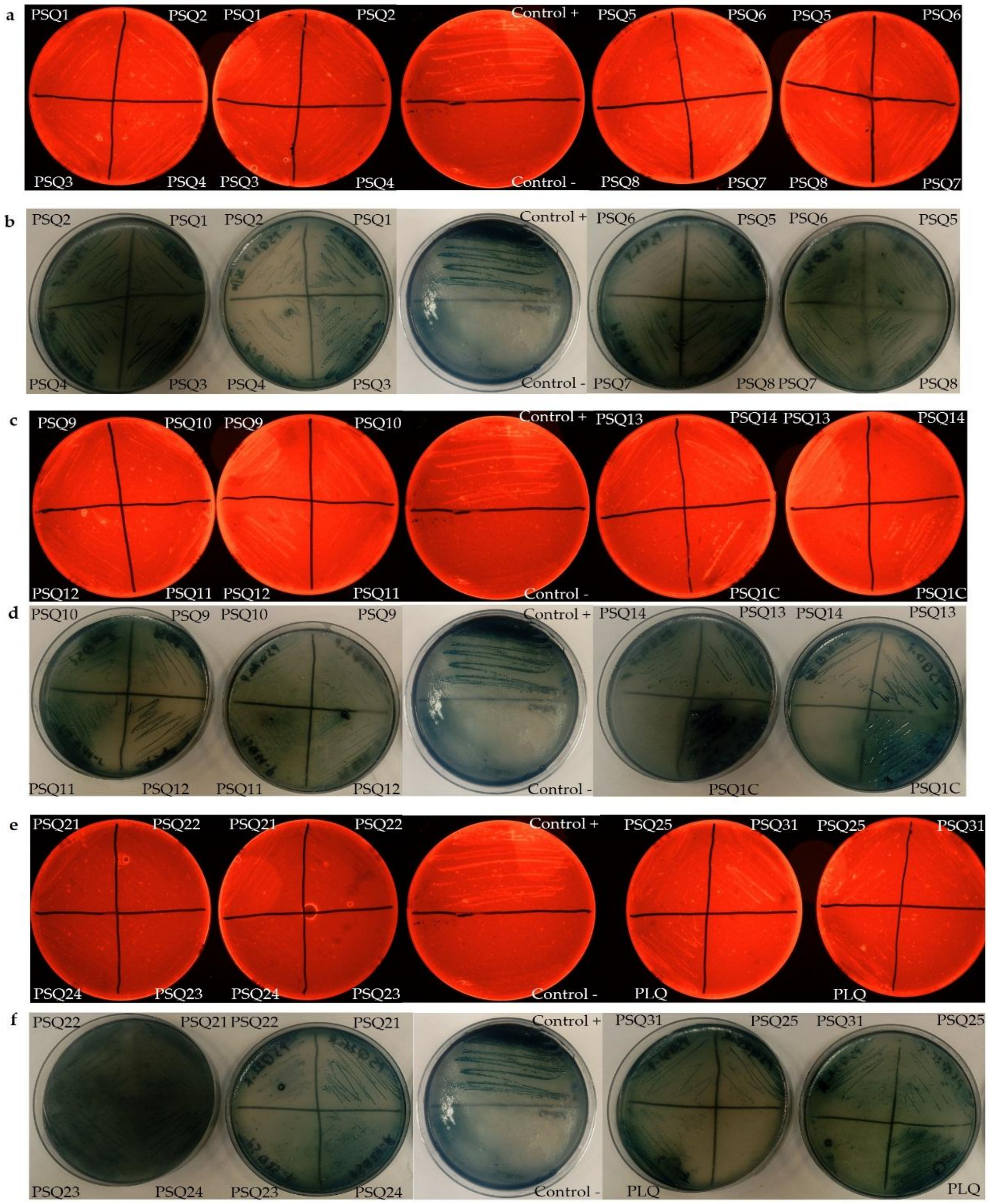

Figure S4. Cells accumulating PHA staining with Nile Red (**a**, **c**, **e**) and Sudan Black B (**b**, **d**, **f**) on agar plates for the strains isolated from the sample S4P-24 (Lake 2) except the strain PLQ (sample S2P-24). Control +: The PHB-producer *Natrinema altunense* strain CEJGTEA101. Control -: The reference bacterial strain *Escherichia coli* DH5α.

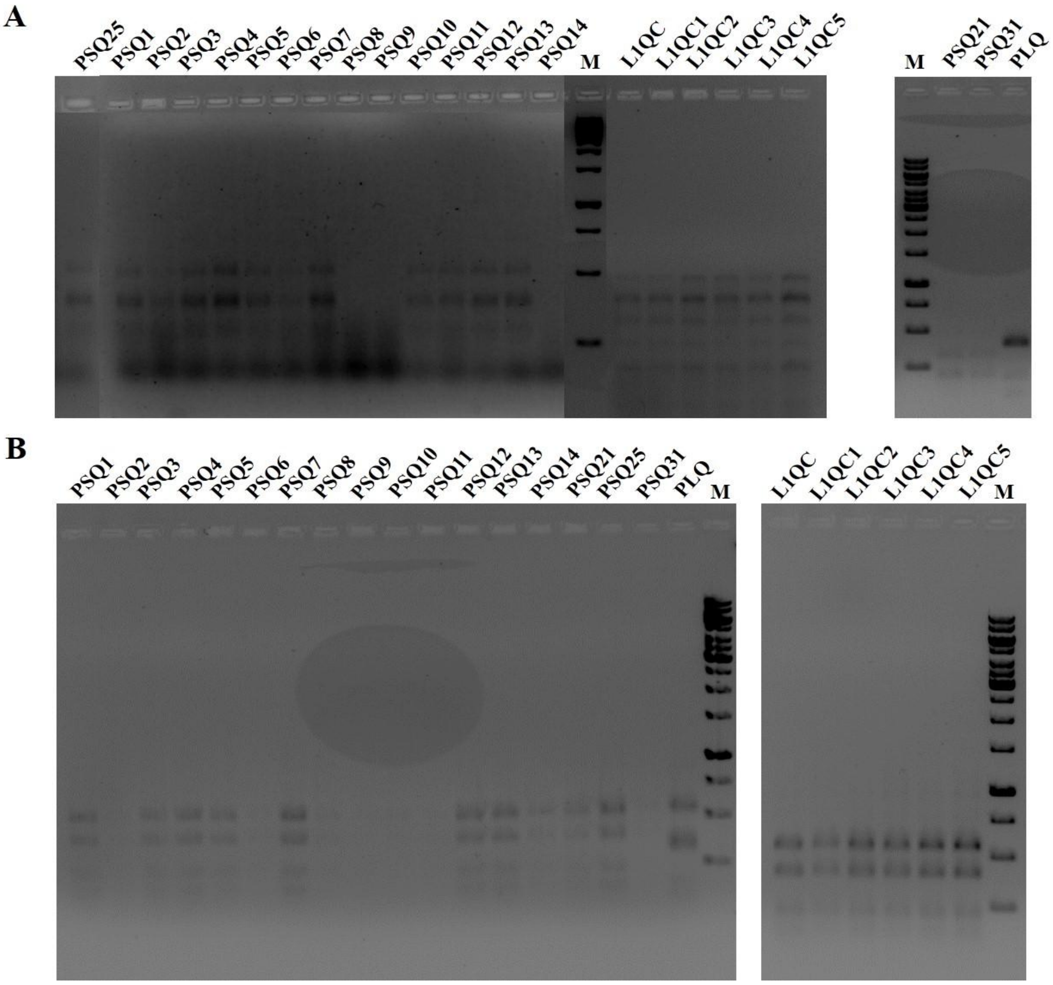

Figure S5. ARDRA profiles obtained by digestion of amplified 16S rRNA of PHA-producing isolates with restriction enzymes *Hae*III (**A**) and *Hinf*I (**B**). Lanes M, 1 Kb DNA marker.

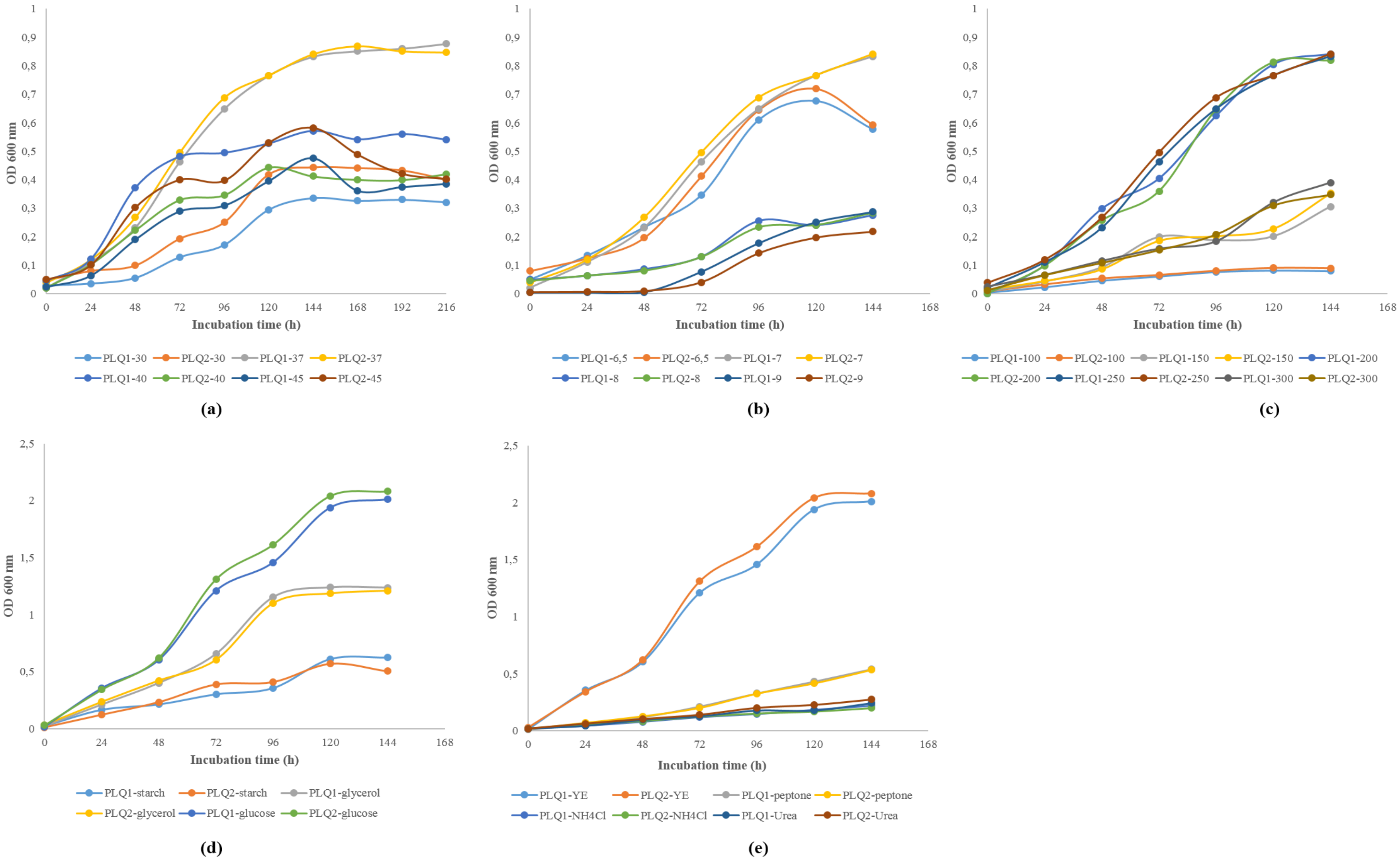

Figure S6. Time course of growth of the isolate *Haloarcula* sp. PLQ at different temperatures (**a**), pH ranges (**b**), NaCl concentrations (**c**), carbon sources (**d**), and nitrogen sources (**e**).

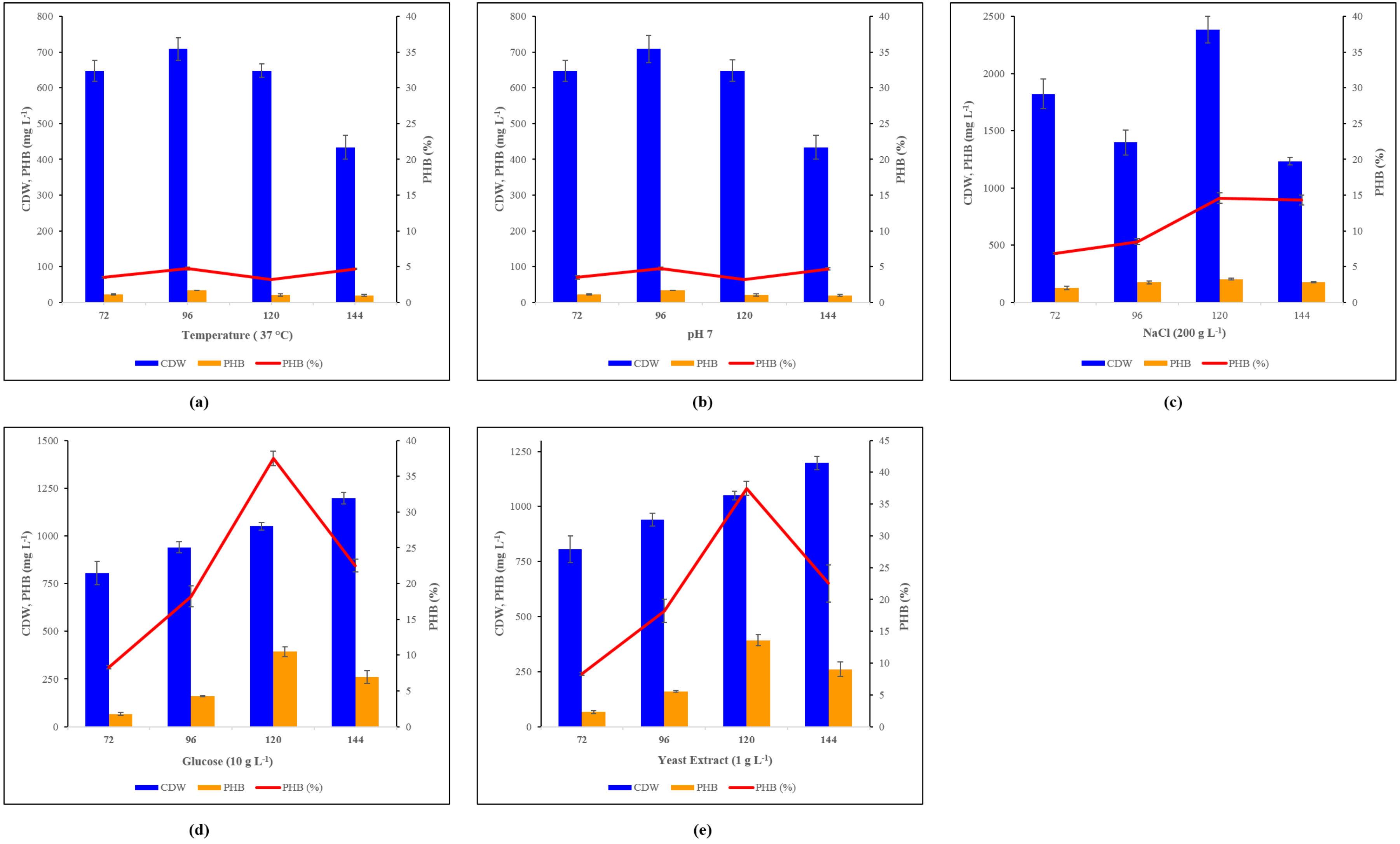

Figure S7. Effect of optimized fermentation parameters on dry cell weight and PHB production of *Haloarcula* sp. PLQ. The data are represented as mean of two replicates ± standard error.

Table S1. Screening of PHA-producing archaeal strains using phenotypic and genotypic methods.

Sample origin	Name of isolates	Morphology	Colony staining method		CODEHOP PCR		Closest phylogenetic relative/accession number
			**SBB**	**NR**	***PhaE***	***PhaC***	
S1G-24	L1QC	cocci	+	+	+	+	*Halostagnicola larsenii*/(PX417682)
	L1QC1	cocci	+	+	+	+	*Halostagnicola larsenii*/(PX417683)
	L1QC2	cocci	+	+	+	+	*Halostagnicola larsenii*/(PX417684)
	L1QC3	cocci	+	+	+	+	*Halostagnicola larsenii*/(PX417685)
	L1QC4	cocci	+	+	+	+	*Halostagnicola kamekurae*/(PX417686)
	L1QC5	cocci	+	+	+	+	*Halostagnicola larsenii*/(PX417687)
S4P-24	PSQ1	pleomorph	+	+	+	+	*Natrinema pellirubrum*/(PX417664)
	PSQ2	pleomorph	+	+	+	+	*N. thermotolerans*/(PX417665)
	PSQ3	pleomorph	+	+	+	+	*N. pellirubrum*/(PX417666)
	PSQ4	pleomorph	+	+	+	+	*N. pellirubrum*/(PX417667)
	PSQ5	pleomorph	+	+	+	+	*N. pellirubrum*/(PX417668)
	PSQ6	pleomorph	+	+	+	+	*N. pellirubrum*/(PX417669)
	PSQ7	pleomorph	+	+	+	+	*N. pellirubrum*/(PX417670)
	PSQ8	pleomorph	+	+	+	+	*N. thermotolerans*/(PX417671)
	PSQ9	pleomorph	+	+	+	+	*N. pellirubrum*/(PX417672)
	PSQ10	pleomorph	+	+	+	+	*N. pellirubrum*/(PX417673)
	PSQ11	pleomorph	+	+	+	+	*N. thermotolerans*/(PX417674)
	PSQ12	pleomorph	+	+	+	+	*N. pellirubrum*/(PX417675)
	PSQ13	pleomorph	+	+	+	+	*N. pellirubrum*/(PX417676)
	PSQ14	pleomorph	+	+	+	+	*N. thermotolerans*/(PX417677)
	PSQ21	pleomorph	+	+	+	+	*N. pellirubrum*/(PX417678)
	PSQ25	pleomorph	+	+	+	+	*N. thermotolerans*/(PX417679)
	PSQ31	pleomorph	+	+	+	+	*N. pellirubrum*/(PX417680)
S2P-24	PLQ	pleomorph	+	+	+	+	*Haloarcula japonica*/(PX417681)

SBB: Sudan Black B; NR: Nile Red. *PhaE, PhaC* genes encoding class III PHA synthase.
The samples were collected from Al Shamal lakes in northern Qatar.

Table S2. Results of the parameters used to analyze the intracellular PHB production in *Haloarcula* cells under optimized conditions.

**Optimized parameter**	**Optimal cultivation condition**	**Average CDW[a] (mg L^{-1})**	**Average intracellular PHB concentrations (mg L^{-1})**	**Average intracellular PHB content (%)**
**Temperature**	37 °C	708.3 ± 31.7	33.6 ± 0.4	4.75 ± 0.2
**pH**	7	708.3 ± 31.7	33.6 ± 0.4	4.75 ± 0.2
**NaCl**	200 g L^{-1}	2383.2 ± 116.8	348 ± 17	14.6 ± 0.72
**Carbon source**	glucose	1050.5 ± 20.3	393 ± 25	37.47 ± 1
**Cultivation time**	120 h	1109.8 ± 58.6	496 ± 24	44.69 ± 2.13

[a] The values were determined by gravimetric method. The experiments were performed in duplicate.
